# Implantation of a totally implanted venous device unexpected discovery of left permanent superior vena cava in a patient with sigmoid colon cancer: a case report

**DOI:** 10.3389/fsurg.2025.1428776

**Published:** 2025-01-21

**Authors:** Shuchen Zhu, Mingyan Dong, Haiguan Luo, Yicui Piao, Qiaohong Zhang, Lihui Huang, Zijie Liu

**Affiliations:** Department of Intensive Care Unit, National Cancer Center/National Clinical Research Center for Cancer/Cancer Hospital & Shenzhen Hospital, Chinese Academy of Medical Sciences and Peking Union Medical College, Shenzhen, China

**Keywords:** persistent left superior vena cava, totally implanted venous device, electrocardiogram-aided tip localization, coronary sinus rhythm, sigmoid colon cancer

## Abstract

Persistent left superior vena cava (PLSVC) is an intrathoracic vascular malformation. Sigmoid colon cancer with a left superior vena cava (LSVC) is very rare. Totally implanted venous devices (TIVADs) are commonly used in recurrent chemotherapy for sigmoid colon cancer. We describe a case of unexpected finding of coronary sinus rhythm in intracavitary electrocardiogram localization TIVAD implantation. Intraoperative ultrasonography revealed a PLSVC.

## Introduction

Implantation of a TIVAD in the PLSVC is very rare. Herein, we present a case of coronary sinus rhythm detected during surgical operation for implantation of an infusion port, ultrasound further revealed a LSVC. We successful implantation of a TIVAD under ultrasound combined with intracavitary electrocardiographic localisation technique in a patient with sigmoid colon cancer and PLSVC.

## Case report

A woman in her late 50 s was admitted to the hospital due to bloody stool and abdominal pain. Colonoscopy biopsy showed tubular adenocarcinoma of the sigmoid colon. The patient had undergone a combined thoracoabdominal CT scan at another hospital before admission. However, the CT did not provide any relevant imaging data, and the CT diagnostic report did not mention the left persistent superior vena cava. According to the pathological type and stage of the tumor, the patients were evaluated before chemotherapy, and the patients' consent was obtained to receive chemotherapy with XELOX regimen. The patient had no previous history of coronary heart disease or diabetes mellitus without chest tightness, chest pain and palpitations. Cycles of chemotherapy are expected to last up to 6 months, TIVADs are effective and safe central venous infusion device. The vital signs of the patient were stable. Electrocardiogram showed normal sinus rate. The *in vitro* measurement method was approximately 14 cm from the expected skin puncture point to the catheter tip position along the right subclavian vein path.

### Surgical procedures

**Figure 1:**
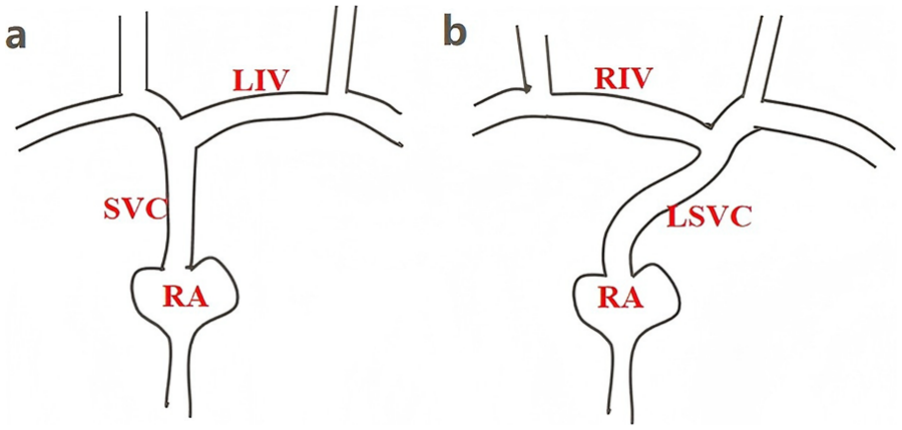
The anatomical diagram of the superior vena cava. **(a)** The normal superior vena cava anatomical diagram. **(b)** The patient's superior vena cava anatomical variation based on B-ultrasound. LIV, left innominate vein; RIV, Right innominate vein; SVC, superior vena cava; RA, right atrium; LSVC, left superior vena cava.

After signed the informed consent, the patient was in a supine position. Under ultrasound guidance, a puncture was made into the right subclavian vein, a guidewire was inserted and the puncture needle was withdrawn. Intraoperative bedside B ultrasound showed that the guide wire direction was from the right subclavian vein into the innominate vein, and no right superior vena cava was found. Intraoperative bedside B ultrasound considered the possibility of left persistent superior vena cava. The diagram of the patient's superior vena cava anatomical variation based on B-ultrasound (Figure 1). A small skin incision was made in the skin around the guidewire, the sheath tube was threaded into the guide wire, The guide wire was pulled out and the catheter was inserted down the sheath tube. Intracavitary electrocardiogram was used for localization, and the *P* wave of electrocardiogram in lead II of the limb was used as the localization wave type. The *P* wave was upright at the beginning of the operation. When the catheter was sent to 15 cm, there was a negative wave at the beginning of the *P* wave, considering the possibility of coronary sinus heart rate. The ideal tip position was withdraw 1 cm from the catheter when the *P*-wave was at its highest point. A 2 cm incision of the skin was made in the right chest wall, and the subcutis was bluntly separated to form a pouch. Tunneled through the pocket and the subclavian vein puncture point, The catheter was attached to a port device along the tunnel. The infusion port of TIVAD was implanted into the subcutaneous pouch, the pouch was sutured. Implantation process of this venous access device takes approximately 40 min.

**Figure 2:**
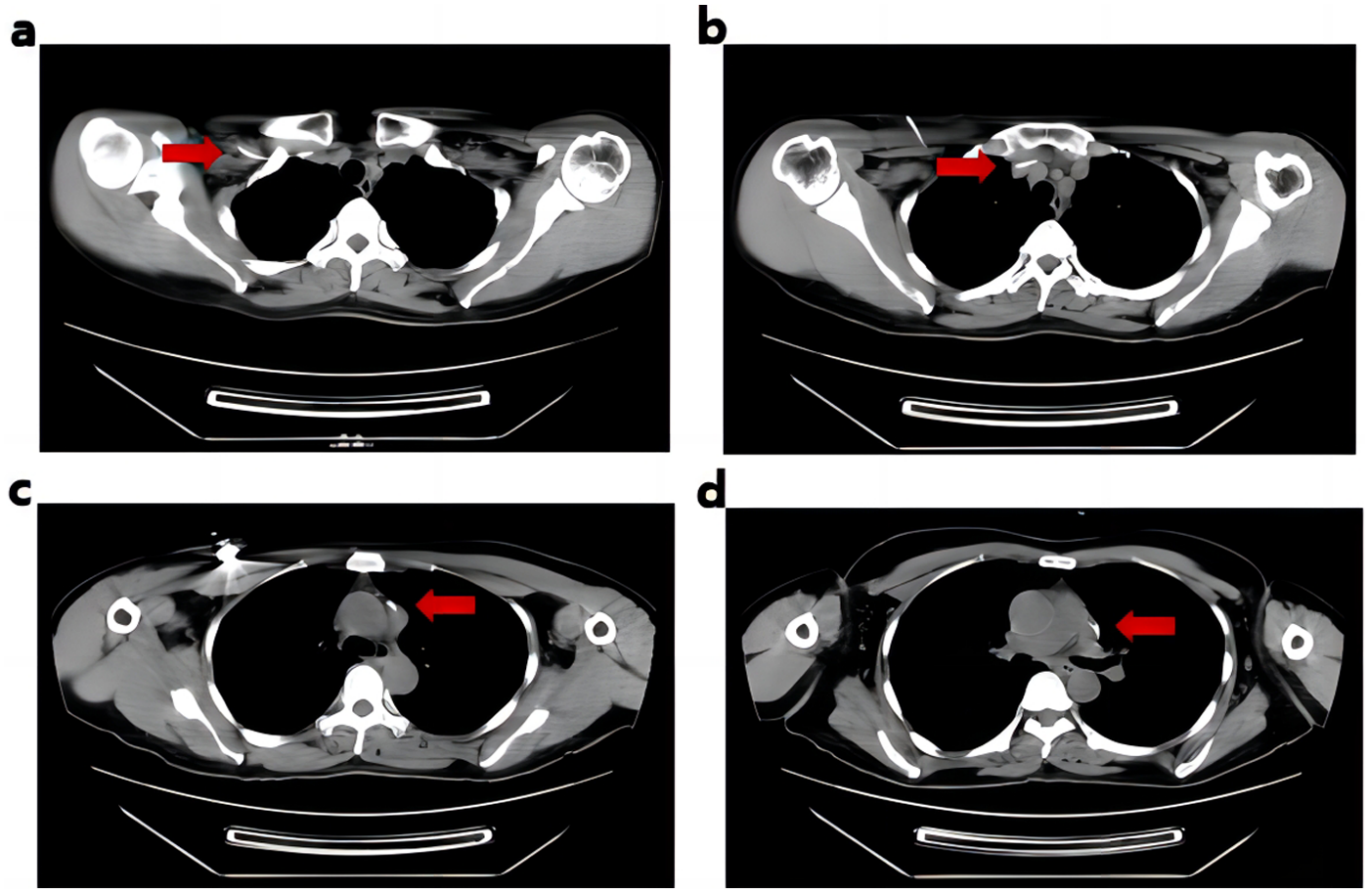
Postoperative CT showed the position of the catheter. **(a)** The catheter was located under the right subclavicle. **(b)** The catheter was inserted into the innominate vein. **(c)** The catheter was entered into the left superior vena cava. **(d)** The tip of the catheter was located at the left lateral level of the lower border of the 6th thoracic vertebra, within the middle and lower 1/3 of the left persistent superior vena cava.

**Figure 3:**
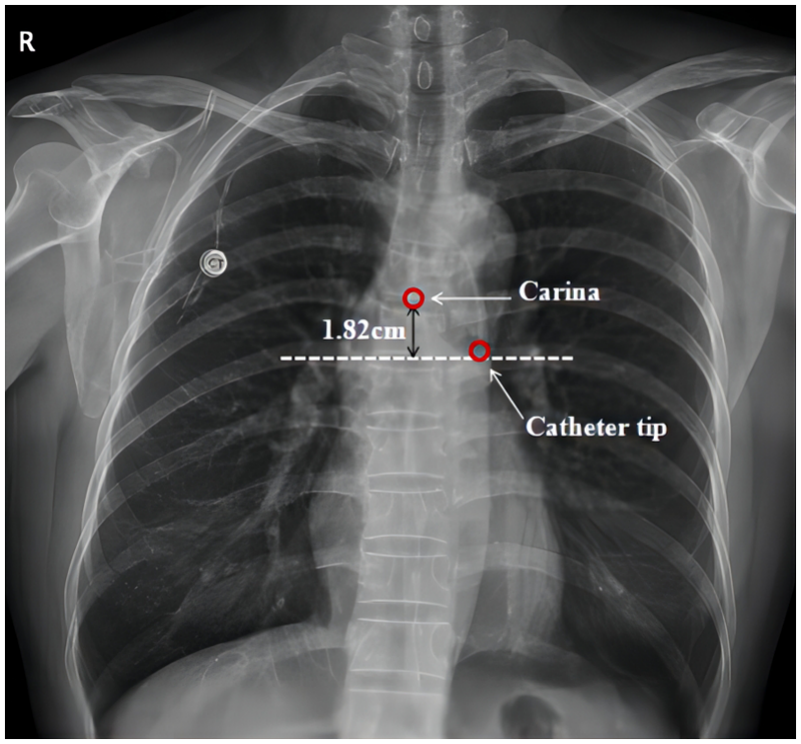
Postoperative chest x-ray showed the catheter tip is located 1.82 cm below tracheal carina and considered the ideal location.

Postoperative chest CT showed superior vena cava malformation: the left and right cephalic and brachial veins converge anteriorly on the left side of the aortic arch, travel down the left anterior mediastinum anteriorly on the left side of the left pulmonary artery, and finally join the right atrium. The catheter reached the left superior vena cava through the right subclavian vein and right brachioceanian vein, and the tip of the TIVAD port was located at the left lateral level of the lower edge of the T6 vertebra (Figure 2). Postoperative chest x-ray showed that the catheter tip was 1.82 cm from the horizontal line of the tracheal carinal (Figure 3), suggested the tip of the catheter was in the appropriate position (1).

## Discussion

PLSVC is one of the thoracic congenital venous malformations, 0.3%–0.5% incidence in normal population (2). The LSVC usually gradually atrophes and regresses with embryonic development, while the PLSVC represents the persistence of the embryonic left anterior main vein. PLSVC mostly flows into the right atrium through the coronary sinus, and most patients have no clinical symptoms and hemodynamic changes. Therefore, LSVC is often accidentally found by ultrasound in the process of central venous catheterization and implantation of cardiac pacemaker.

The PLSVC was classified according to the absence of the right superior vena cava and its adjacent anatomy of the central vein. Type I: Anatomy of the superior vena cava in the normal heart (3); Type II: presence of LSVC with absence of right superior vena cava, accounting for 18% of PLSVC patients (4); Type IIIa: there were LSVC and right superior vena cava at the same time, and there was an unnamed vein connection between the two sides of the superior vena cava, accounting for 60% of the PLSVC population; Type IIIb: LSVC and right superior vena cava were present at the same time, and there was no innominate venous connection between the two superior vena cava (5). In this case, the imaging after the implantation of the TIVAD could confirm that the catheter was placed in the LSVC, and the right superior vena cava was absent. The blood from the right jugular vein and right subclavian vein entered the LSVC through the innominate vein, which belonged to type II PLSVC. We chose TIVAD because it is safer and more convenient than PICCs and CVCs (6). PLSVC must have a sufficient internal diameter, a diameter ratio of catheter to PLSVC > 0.45, sufficient blood flow, and no resistance to pullback through the catheter to reduce the incidence of thrombosis.

In type II PLSVC, due to the absence of the right superior vena cava, the blood of the right jugular vein and subclavian vein flows into the LSVC through the innominate vein, and there may be a corner at the confluence of blood vessels, so the choice of the left internal jugular vein is better than the right internal jugular vein and right subclavian vein. For this patient, the inner diameter of the left internal jugular vein and the left subclavian vein is smaller. The inner diameter ratio of catheter to vein was less than 0.45, and the patient's right subclavian vein was thick, and there was no resistance when the guide wire and catheter were placed, so the right subclavian vein was selected for puncture. Because PLSVC patients have abnormal blood vessels, they often mistakenly think that the catheter is inserted into the aorta or the catheter has broken through the vein and entered the mediastinum during the operation. The operator can feel the pressure and the color of the blood returned by the puncture needle to identify the arteries and veins during the operation. Blood gas analysis was compared between the arterial end and the infusion port. The intracardiac electrocardiogram-guided catheter tip positioning has been widely used in recent years due to its accuracy, safety, and efficiency (7). Most PLSVC drains into the right atrium through the right coronary sinus (8), The tip of the catheter should be located at the middle and lower third of the left persistent superior vena cava to the opening of the coronary sinus. If the tip is too shallow, the blood flow will be insufficient, the local turbulence will increase the possibility of thrombosis. On the contrary, if the tip is too deep, the turbulence will stimulate the coronary sinus and induce arrhythmia.

Changes in electrocardiographic positioning of the catheter tip under normal anatomical conditions: *P*-wave changes in the limb II lead intracavitary electrocardiogram are used to guide the position of the catheter tip. When the catheter tip enters the superior vena cava, the *P*-wave in the II lead begins to increase gradually as the catheter tip penetrates deeper and deeper, and the *P*-wave amplitude is the highest in the cavoatrial junction (CAJ), which is the ideal position of the catheter tip, and then it passes through the CAJ point and enters the right atrium, and then negative *P*-waves appear. After crossing the CAJ point and entering the right atrium, negative *P* waves appear, and it is generally believed that the catheter tip reaches the ideal position when the P/R amplitude ratio reaches 50%–80% (9). In this case, the change of *P* wave in lead II was detected by intracavitary electrocardiogram. With the assistance of bedside B ultrasound, when the tip of the catheter entered the left superior vena cava, a negative wave appeared at the beginning of the *P* wave in lead II, presenting as bidirectional *P* wave. When the absolute value of the *P*-wave amplitude was maximal, it was withdrawn 1 cm, and intracavitary electrocardiogram mapping at this time showed that the absolute value of *P*/*R* amplitude ratio was about 60%. The final postoperative chest CT showed that the tip of the port was located at the left lateral level of the lower edge of the 6th thoracic vertebra, which was at the middle and lower 1/3 of the superior vena cava. The preoperative electrocardiogram of the patient with left persistent superior vena cava showed sinus rhythm, but the changes of *P* wave in lead II of the limb were different from those of the normal heart during the operation. In this patient, the LSVC drained into the right atrium through the right coronary sinus (confirmed by postoperative imaging), and the right coronary sinus was dilated. In the resting state, the excitability of the sinus node was the highest, so the preoperative diagnosis was sinus rhythm. When the catheter tip was stimulated into the coronary sinus, the rhythmicity increased and the atria were agitated retrograde, and abnormal *P* waves appeared. Clinically, the electrocardiogram shows inverted *P* wave in II, III, aVF, *P*-*R* interval > 0.12 s, normal QRS duration and ST-T, which is called coronary sinus rhythm. Coronary sinus tachycardia accounts for about 3% of focal atrial tachycardia (10).

Regular follow-up: In addition to the routine health education of infusion port maintenance, the patient also received health education on the related knowledge and possible complications of PLSVC. The patient returned to the hospital regularly every month for maintenance, and 100 u/ml heparin sodium was given to seal the tube. The patient has completed chemotherapy, and ECG monitoring was conducted during chemotherapy, and no malignant arrhythmia events occurred. The patient had been indwelling venous access port for 1,108 days, without chest tightness, chest pain, palpitation and other manifestations. The venous access port was unobstructed, and the regular electrocardiogram showed sinus rhythm and normal electrocardiogram.

## Conclusion

PLSVC is rare and clinically asymptomatic. It is often found accidentally during invasive operation of deep veins, and catheters can be implanted in LSVC with good preoperative assessment. During the operation and treatment, we need to closely monitor the patient's heart rate and blood pressure, and pay attention to the occurrence of malignant arrhythmia. The application of bedside ultrasound combined with intracavitary endocardial electrodes localisation technology can improve the success rate of catheter placement, determine the direction of the catheter, and efficiently and accurately adjust the position of the tip of the catheter during the operation to reduce the possibility of the operator and the patient being exposed to x-ray radiation, and to shorten the duration of the operation, which is a very great advantage if the endocardial electrocardiography in the operation is not used. If intracavitary electrocardiogram suggests the possibility of coronary sinus rhythm, it is necessary to be alert to the occurrence of PLSVC.

## Data Availability

The original contributions presented in the study are included in the article/Supplementary Material, further inquiries can be directed to the corresponding author.
